# Multiple Grammars and the Logic of Learnability in Second Language Acquisition

**DOI:** 10.3389/fpsyg.2016.00014

**Published:** 2016-02-04

**Authors:** Tom W. Roeper

**Affiliations:** Department of Linguistics, University of Massachusetts, AmherstAmherst, MA, USA

**Keywords:** multiple grammars, learnability, transfer, acceptability/grammaticality judgments, minimalism, verb-second, interfaces

## Abstract

The core notion of modern Universal Grammar is that language ability requires abstract representation in terms of hierarchy, movement operations, abstract features on words, and fixed mapping to meaning. These mental structures are a step toward integrating representational knowledge of all kinds into a larger model of cognitive psychology. Examining first and second language at once provides clues as to how abstractly we should represent this knowledge. The abstract nature of grammar allows both the formulation of many grammars and the possibility that a rule of one grammar could apply to another grammar. We argue that every language contains Multiple Grammars which may reflect different language families. We develop numerous examples of how the same abstract rules can apply in various languages and develop a theory of how language modules (case-marking, topicalization, and quantification) interact to predict L2 acquisition paths. In particular we show in depth how Germanic Verb-second operations, based on Verb-final structure, can apply in English. The argument is built around how and where V2 from German can apply in English, seeking to explain the crucial contrast: “nothing” yelled out Bill/^*^“nothing” yelled Bill out in terms of the necessary abstractness of the V2 rule.

## Introduction^1^

Modern Minimalism (Chomsky, 1995, 2013) has made grammatical description dramatically more abstract. If it is on the right track, then it should reflect typical scientific progress: it should both transparently capture deeper rules and make more refined predictions about grammatical detail (Adger and Smith, 2005).

We argue that Minimalism naturally forces an approach to every speaker's grammatical knowledge in terms of Multiple Grammars (MG), which in turn enables sharp predictions about second language acquisition (L2) to arise. Thus, another virtue of modern minimalism is that it invites a new domain of data to be directly relevant to linguistic theory—and, we may reasonably expect, to pedagogical practice.

Unlike, for instance, Phrase Structure Rules, which are stated in language particular terms, we can state rules in terms of Merge, Labeling, Feature-satisfaction. When the rules are stated as abstractions in terms of abstract minimally labeled categories, they immediately apply to many languages. It follows that a speaker of L1 can—and I argue must—apply these rules to L2.

This perspective leads to a little logic which is the backbone of this essay (Roeper, 2011, 2014):

(1) a. Grammars must be simple. They are not encumbered with notation for choice and optionality.
b. Principles of grammar are very abstract.
c. Such abstract grammars will inevitably apply to many languages, not just one.
d. All speakers will possess multiple rules, in effect Multiple Grammars, which must automatically be applicable in a second language.
e. If true, then the study of L2 acquisition (and variation in general) provides a unique domain to test whether these levels of abstraction are appropriate.

Language typology suggests that Universal Grammar (UG) captures a number of language families with simple alternations: VO or OV, Wh-movement, or non-wh-movement, Scope inversion or surface scope, Pragmatic Object deletion, or not (Hot/Cool distinction) It is natural to expect that every speaker will have all prototypes available—especially, but not exclusively, if they speak an L2 that carries them.

This approach, in turn, can be viewed as a version of Transfer (Full Access, Full Transfer), common in the literature and assumed by many (see White, 2003 for an overview). However, it does not entail that a rule of one grammar is actually transferred into another grammar. Why not? Because the simplicity of rules would not allow a rule to be stated with all the contingencies that are found in every particular grammar. For any particular language, many specific lexical contingencies and structural variations are involved, as we shall detail, and they exhibit the over-application and under-application of rules that is typical of the variation found in L2.

In sum, MG theory follows from both the spirit and substance of the Minimalist approach. Fortunately, the grammatical subtlety of work in L2 has matured to the point where we can begin to develop a Learnability Logic and predict cross-linguistic L2 acceptability judgments under the assumption that more than one grammar is simultaneously available^2^. The goal here will be to show why and how such logic should work, examine cases where it applies, and predict L2 acceptability/grammaticality judgments. In other words, to seek theoretically predictable “grammaticality” from an L2 perspective in the same manner we seek to predict ungrammaticality in typical theoretical work.

Many of our arguments are implicitly present in a number of recent papers that advocate Full Access/Full Transfer, Feature-Reassembly, and Variational Learning (for which MG theory is a prerequisite). Our goal is to take a few steps—just a few—toward the technical precision found in monolingual generative studies.

### Overview

The essay is focused on a detailed discussion of an abstract version of V2, formulated with open XP and YP environments:

$$\begin{array}{l} V2\;Rule:XP\;YP\;V=>XP\;V\;YP.^{3}\\ \;1\;2\;3\;1\;3\;2 \end{array}$$

This leads to predictable overapplication of rules in Wh-movement, Quotation, Topicalization, particle behavior, and potential interactions with Information Structure, which can then influence L2. This in turn may yield greater insight into V2.

Section Avoiding the Concept of Transfer considers Dimensions of Transfer and Compatibility among Modules. Section Diverse V2 considers V2 in terms of different Force types (Wh-movement, Quotation, Topicalization) and an extensive discussion of particles. Section Moving Toward L2 Formalism contrasts V2 I L1 and L2 environments, arguing that the acquisition path of L1 reveals the right level of abstraction. Then in Section Moving toward L2 Formalism we consider Information Structure and Discourse, and the Principle of Minimal Modular Contrast, and the role of Exhaustivity in Topicalization. The next Section considers Missing Subjects and Objects, and then considers Dialects and Modular Incompatibility. Then we return to a more extensive discussion of and integration with MG theory with Variational Learning, Full Transfer and Full Access, and Feature Re-assembly. Section Conclusion, concludes the essay with a summary of why Minimalism makes particular predictions about L2.

## Avoiding the concept of transfer

The term “Transfer” has been pivotal in much of the discussion but in my view it both overstates and understates how L1 influences L2. In one sense, the MG approach builds on the core intuition of Transfer, but in another sense our goal is to argue against Transfer as the core concept and dissolve the term into concepts that reach further into making predictions based on UG for L2 acquisition. If separate grammars are simultaneously present in speakers, the status of these two grammars will vary with how far advanced L2 knowledge has developed. And it may affect comprehension and production quite differently^4^.

### Transfer dimensions

What is Full Transfer? Although the term is widely used, it is not always clear what the status is of something transferred in each respective language. One extremity seems to be: If one fully Transfers part of one grammar into another, then it is a compatible and unrecognizable part of that language. In effect, one language incorporates or copies part of another. That is true of the many words borrowed from one grammar into another. Speakers are often unaware of their origins. They are not present in a second grammar in the speaker's mind. Charm came from French, but is pronounced by English rules, and it does not entail the presence of a French grammar in an English speaker.

In fact Full Transfer would entail many special statements with respect to other modules: case, agreement, thematic roles, wh-movement, LF movement. The requirement on Simple Rules aims to avoid exactly this complexity. We will advance a general proposal that limits the interaction of modules below called: Minimal Modular Contact. It, obviously, remains a programmatic goal, but it can guide research nonetheless.

### Transfer induced ambiguities and modular compatibility

What is a paradigm case of Transfer? Consider (2)

(2) the dog chased the cat

It is either SVO or OVS (via V2 on SOV) in several Germanic languages, but only SVO in English. Yet speakers of those Germanic languages demonstrably (as we discuss below) register the other meaning. Under a Multiple Grammars (MG) approach^5^ the argument is not simply that L1 is used in an L2 environment. Such examples obey what we can call:

(3) Complete Modular Compatibility

That is, no other module of L2 is disobeyed as in (2)^6^. So (3) predicts that for instance, V2 can be applied in sentences to create OVS structure just in case, the Subject-verb AGREEMENT module is not violated, as it would be below (4):

(4) the dogs are chasing the cat = > ^*^dogs = object.

When this criterion is imperfectly observed, as it is sometimes, then we predict degrees of acceptability of MG in L2 (an issue raised by a reviewer) depending upon the status of the Agreement module in a grammar. This occurs when there is a variable state of L1 and L2 within individual speakers. How could there be a variable status of a module? German, for instance, has a very complex two-layered system of case-assignment and agreement. If one had only the Strong system or only some of the Agreement paradigm, then the appeal of a rule without agreement is stronger.

So we predict, if favored by pragmatics, a first stage German or Dutch learner of English (which happens, see below) might indeed allow: the rats are chasing the dog to mean the dogs chase the rats, while the advanced learner, as evidence below suggests, will inevitably continue to allow only (2) to be misanalyzed in comprehension when Subject-verb agreement is unmarked.

Thus, it captures what can be descriptively observed as a moment of L1/L2 Non-interference:

(5) L1/L2 Non-interference:
Rule X from L1 can apply in L2 because
a. no L2 module is violated.
b. no obligatory L1 module is ignored.

This is a perfect case where we argue that nothing has been “Transferred,” but rather L1 simply operates in L2. The same holds for the application of Inverse Scope for quantification in an L2 with only Surface Scope where, for instance, the Case-assignment module would make no difference. Similarly, if the Interface with Logical Form is universal, then it does not have to be learned or Transferred, which we discuss. Nonetheless, we argue that within L2, L1/L2 non-interference can make surprisingly subtle predictions. We show where the grammar of separable particle constructions in German could be involved in the L2 analysis of English particle behavior in quotation.

In sum, MG theory is a significant form of grammatical economy: information is not written twice, but rather a single representation is accessible in more than one language. We expect that every grammar will contain simple pieces of UG that are not exclusive to it, but linked to another grammar type, hence Bilingualism is universal^7^.

The critical reader might point out that this approach is akin to the claim that there are Linguistic Universals that can be innate and therefore do not have to be separately stated. Indeed MG theory is really an extension of traditional UG assumptions.

### Is genie relevant?

One intriguing question can be asked (as a reviewer did): does one access UG through L1? The answer should be “no” because a speaker should still have access to UG as a set of inborn options, such that he could, for instance, set the pro-drop parameter the opposite way. However, it does seem that L1 is necessary to trigger the availability of UG under the Critical Period hypothesis.

If that were not the case, then Genie (Curtiss et al., 1974), the 12 year-old child discovered without a first language, would have learned English just the way that any L2 learner of English would in high school. But she was unable to. So we would argue that L1 is necessary to trigger UG, but not that one must “go through” L1 to locate UG or basic rules.

## Diverse V2

We will now examine a series of less frequent constructions in English where V2 might apply.

In “Universal Bilingualism” (1999), I argued that English Quotation should not be collapsed with other forms of inversion into a complex rule (such as the version in Collins and Branigan, 1997) but remain a UG option selected by both English and German in a form that remains simple, following a basic principle of Avoid Complex Rules (see Amaral and Roeper, 2014 for more specifics). We will examine among other constructions:

(6) Wh-movement
Quotation
Topicalization
Empty Objects
Auxiliary inversion

### V2 and Wh-questions

Could V2 be present in Wh-questions? Rankin (2015) suggests that L1 may be *covertly* applied and rejected unconsciously quite frequently in the process of comprehension. This leads to unseen labor from L2 speakers in many grammatical situations. These are often assumed to be “processing” difficulties, but I consider them instances of representational conflict between two grammars. The comprehension dimension may exist even when a Speaker knows to avoid V2 in production.

Rankin (pc) suggests, based on judgment data, that there appears to be a difference in how L1 German learners of English comprehend these two sentences (in ongoing work):

(7) a. who woke up John
b. who woke John up.

German speakers allow who in (7b) to have an object reading (John woke who up) while in (7a) who has a subject reading (someone woke up John). Both sentences involve movement of the Main Verb, while one moves the particle as well (7a). Only (7a) receives a purely English analysis (John = object), while (b) allows post-verbal John to be subject.

The implicit reasoning seems to be this: German disallows a particle to move with its verb (8d), while it is optional in English (8a,b):

(8) a. John woke up Bill
b. John woke Bill up
c. Hanns weckt ihn auf.
[John woke him up]
d. ^*^Hanns weckt-auf/aufweckt ihn.
[John woke up/upwoke him]

Therefore, the fact that verb+particle moves as a whole (8a), signals that English grammar is used, because that is impossible in German. Consequently movement is to the TP, leaving the subject in SPEC, which guarantees that when who is moved to CP, it remains a subject in (7a). Had the object moved in English, then Tense would move to C where it would have forced do-insertion:

(9) who did John wake up?

But if only the Head verb (wake) and the wh-word both move to CP, as in German (8c), then John could be the subject left in IP in who woke John up leading to the evident miscomprehension.

The unseparated movement of verb+particle blocks access to V2 from the German-L1 speaker's perspective. There is no verb in English ^*^to upwake, therefore it must be movement to the English permitted position. Once again, the other case (10) meets the Non-interference Identity criterion:

(10) who woke John up?

(10) could be either (a) movement of wake to T or (b) movement to C, with no other modules disturbed in *either* case. Hence the German speaker cannot restrain the V2 reading in comprehension. These results hold for advanced speakers, Rankin argues, and therefore are not typical production errors, but they show the continued presence of MG.

### Quotation

From another angle, exactly the same contrast is at work in quotation as Amaral and Roeper (2014) show based on Bruening (2013), Collins and Branigan (1997), and Alexiadou and Anagnostopoulou (2007). If V2 applies in English, as (11) suggests, then it will allow the verb+particle to move to C:

(11) a. “nothing” yelled out John

and no do-insertion is allowed:

b. ^*^“nothing” did John yell out.

Of greater interest here, is the fact that in English the particle cannot be left behind (12a) while in German it must be left behind (12c):

(12) a. ^*^“nothing” yelled John out.
b. ^*^ “nichts” ruft aus/ausruft Hanns.
[“nothing” yelled-out/out-yelled John]
c. “nichts” ruft Hanns aus.
[“nothing” yelled John out]

The German facts are not surprising and lead to a prediction. If questioned, German speakers should find (11b) acceptable (which my informal exploration supports) because only the Main Verb, that is the Head, not the particle can move under V2 in German, which is in line with other constraints on Head-movement.

It is important to note that only verb+particle moves over the subject. It is not the case that any larger VP (said to Bill, screams angrily) can move over the subject in English as these data show:

(13) a. ^*^“no” said to Bill John
b. ^*^? “yes indeed” screamed angrily Fred

Therefore, the constraint to Head-movement which characterizes V-to-I movement in many grammars is upheld. The fact that verb+particle must move to C in quotation, using V2 and disallowing a stranded particle, calls for a more refined interpretation of the interaction of V2 and separable particle verbs. If the use of V2 in English is only stateable at an abstract level, then it will not be able to access the separable property of particles in the English lexicon, and therefore the whole verb+particle moves. If true, we might expect to find the restriction elsewhere. We turn now to other restrictions on particle movement which enlarge the question.

### Topicalization

While German allows Topicalization of almost any element, precisely particles are excluded in both English and German?

(14) a. English: ^*^out spread John, ^*^over came Bill
^*^out cried he, ^*^up threw Mary, ^*^on carried Fred
b. German: ^*^Aus ruft er.
[Out yelled he.]
“He yelled out”^8^

Why? It is noteworthy that there are subtle differences here which point to the question of what the exact Label on the particle is:

(15) a. ^*^über läuft er [^*^over ran John]
b. ‘rüber läuft er [here over ran John]

(15b) is a reduced form of an adverb phrase herüber läuft er (here-over ran he), via a morphological rules that adds her-.

The same holds for English with stylistic inversion:

(16) over here came Bill.

and we would predict that these judgments would hold across L2-English and L1-German, but they deserve examination.

These judgments obey the underlying abstraction that, while verb movement involves just Heads, movement to a Topic position must involve a phrase, a Maximal Projection. The Label on a particle is not absolutely clear, but it is evidently not projectable to a Maximal Projection.

### Particle mystery

A deeper question is why we can leave the particle behind with movement to TP:

(17) John yelled the answer out.
[likewise: shout out/scream out/holler out/bellow out]

but not with movement to C in English?^9^ Interestingly Quotation does not allow particle-stranding either, even when movement is clearly to TP (18a):

(18) a. ^*^ John hollered “I can't come” out.
b. John hollered out “I can't come”^10^.

And this holds for passive as well:

(19) ^*^“I can't come” was hollered by John out

although one can have the by-phrase move over indirect objects (20a), just not the particle (20b):

(20) a. Presents were given by Bill to John
b. ^*^Presents were given by Bill out.
c. Presents were given out by Bill

And this same constraint applies to expletive cases like:

(21) a. there walked a man in/ ^*^there came a man over.
b. there walked in a man/there came over a man.

And, finally, an old puzzle about particles remains:

(22) a. John threw Bill down an apple
b. ^*^John threw Bill an apple down.
c. he read me out the riot act
d. ^*^he read me the riot act out.
(23) a. He tossed up these apples to the kids, but he could not toss them up those.
b. … ^*^toss them those up.

In (23b) there is no hint of Heavy-NP shift at work. We conclude that something much deeper, still unknown, is at work here.

## Moving toward L2 formalism

An important question buried in the discussion is this: if we have access to L1 in L2, then when is it operative? We have argued, as others have (Westergaard, 2003) that when full Non-interference Compatibility is present, then the application of L1 is unstoppable at the comprehension level. Where refined features of a structural description must be accessed—then the application is less automatic and less under speaker control, as in the examples just mentioned.

This can be discussed in somewhat more formal terms. Whatever allows the intermediate appearance of a particle must be stated over a full Structural description (to use older terminology, Chomsky, 1957):

(24) NP2 NP1 verb+particle = > verb NP2 NP1 particle ⇒
verb NP2 particle NP1 *trace*.

Because we have a rule of Heavy-NP-shift one might imagine that it is a subpart of that rule which is expressed specifically when particles are present.

One might also advance the view that a local phonological operation is involved, so that it is very narrow inversion. But exactly this suggestion would not explain why one cannot move over a simple subject:

(25) ^*^ “no” screamed Bill out.

Altogether these facts point to the conclusion that it is only over a single object that one can move a particle verb^11^.

One reason that I explore this mystery here is that the behavior of L2-speakers might easily supply clues about the right level of abstraction that is relevant for the L1 description. In fact, numerous controversies over the ideal rule in a given language might be resolved if we treat the L2 data as pertinent to the original grammatical description, rather than assuming that L2 exploration should only proceed where the L1 is well-understood.^12^. Will German L2 speakers of English accept or reject this entire array of facts about stranded particles? If they apply German V2 to English, then the first hypothesis is that all the stranded particle sentences should be acceptable. It is difficult to reason further until we have the evidence.

Since this domain is ripe for seeking L2 judgments, let us make some predictions about judgments of Germans learning English:

(26) 1. German speakers would find it fine to say:
a. (20b) “nothing” yelled John out.

because they invoke the requirement of separability for prefixes.

They will easily comprehend and produce:

b. “Nothing” said Bill

which has no particle and is pure V2. They will comprehend, as extended V2, but perhaps resist producing:

c. “nothing” yelled out Bill.

because the particle should be and can be left behind in German.

2. Both English and German speakers will reject:
a. ^*^over came Bill

because neither language allows Topicalization of a particle, but both will accept:

b. over here came Bill

because it has been Relabeled in the lexicon as an Adverb. In this case, we predict that the idiomatic form in English (which violates the constraints):

c. in walked John

will be rejected by German speakers because it is treated as a separable prefix (eingehen).

How does (c) become acceptable in English? Suppose we argue that it can undergo Relabeling as an Adjunct Adverb in English, which is impossible without a further morpheme in German (ein = > herein). Is there a theoretical implication here? Is idiomaticity the full explanation here? It could be that Relabeling in the lexicon from particle to adverb requires additional morphology in German but not English. Therefore, the German speaker cannot relabel the bare particle in, because it would require Relabeling via a morpheme, which is available in the lexicon, but cannot be accessed in the syntax. A deeper principle could be involved: L1 cannot apply to L2 if it would entail a rule that crosses an Interface^13^. Another prediction is that all who reject (b) will also reject (c).

Further predictions:

3. German speakers will allow double objects in English
a. ^*^he read me the book out

since analogous forms are acceptable in German

b. “Er hält mir nie die Tür auf” (Google)
[he holds me never the door open]

Now we can speculate about why it should be acceptable. In English, following Keyser and Roeper (1992), the dative and particle occupy the same position, which blocks even isolated dative objects:

b) he yelled the answer out to me.
^*^?he yelled me out the answer.

while in German a productive benefactive dative pronoun exists which could be used in English (also available in English dialects). This makes the further prediction that a full nounphrase might be blocked for the German speaker:

(27) ?He yelled John the answer out.

One might regard this as a highly local point of “transfer.” Instead we can sketch the following argument: suppose that using the German benefactive dative in English is a late adjunction in the derivation of the sentence and therefore it satisfies the Compatibility criterion.

If German speakers “accepted” this sentence, but never would use it, then it becomes a clear example of allowing an alternate German grammar to operate in English, not a “transfer.”

These predictions call for careful study, but the cross-linguistic reasoning should be clear.

The upshot here is again that verb+particle behavior remains an unsolved mystery^14^. The larger array of data suggests that the simple V2 rule is involved with a further constraint consistent with the formulation of abstract rules:

(28) Apply rule to the highest possible Projection

This would favor not separating verb and particle and therefore taking a V node that would not see a division between verb and particle.

### L2 path and the L1 path

One angle from which to examine the formalism is to ask:

Does L2 acquisition mirror the L1 path?

Amaral and Roeper (2014) argued that V2 is acquired piecemeal in L1, resolving a disagreement between Wexler (1998) and Yang (2002). Wexler (2011) argued that children acquired V2 very early because forms like:

(29) da geht er [there goes he]

occurred very early in the 2 year range, while Yang argued that V2 was not acquired until very late because object movement did not arise until 4 years^15^ in children, nor was it frequent in the input:

(30) Fleisch isst er [meat eats he]

In the adult grammar, V2 is expressed with respect to an abstract maximal projection, XP. Evidence of late OVS from Yang but early LOC-V-S suggests that children proceed through a series of specified XP's before they generate the full abstract rule, which no one to my knowledge has thus far carefully traced:

(31) Typical forms of V2:
a. Subject NP: **Er** frisst Fleisch.
He eats meat.
b. LOC: **Da** sing er.
There sings he.
c. ADV: **Schnell** fährt er.
Quickly moves he.
d. DO: **Fleisch** frisst er.
Meat eats he.
(32) Less discussed forms of V2:
a. Quotation: **“Willkommen”** sagt Herr Anders.
“Welcome” said Mr Anders.
b. VP fronting: **Zu mir allein kommen** will er nicht.
To me alone come wants he not.
“He does not want to come alone to me.”
c. Empty Topic in Discourse:
Wo ist das Fleisch? **__** frisst Hanns schon.
Where is the meat? __ ate John already.
d. Conjunction:
Hanns spielt oft, **so** kann er ohne Mühe uns helfen.
Hanns plays often, so can he without difficulty us help.
“Hanns plays often, so he can help us without difficulty.”

### Left-dislocation option

One piece of evidence from Roeper (1973) is that children seem initially unable to carry out the rather rare VP-fronting operation. Thus, among roughly 40 children at the age of 4 years, most repeated:

(32a) Fussball zu spielen macht Spass
[to play football makes fun]

as:

(32b) “Fussball zu spielen, das macht Spass”
[to play football, that makes fun]

adding a resumptive pronoun with left-dislocation. By contrast, without zu the children rarely inserted das:

(32c) “Fussball spielen macht Spass”
[football-playing makes fun]

Why? Because the compound constitutes a typical DP while a fronted VP evidently does not fall under a DP label, therefore a DP equivalent (das) must be added, converting the structure into Left-dislocation. Adults allow V2 after any fronted XP, but only when the XP abstraction has been fully projected.

These data indicate with some subtlety the precision with which the V2 rule that children use must be represented. They had not yet fully extended the representation of V2 to have a lefthand XP. They must still be assembling particular local environments, not yet collapsed into a single rule.

Data of this kind is what we need to see at what formal level the rule is being written in the child's grammar. The exact steps in the abstraction process would be good to know because we could then determine if they are repeated in L2. For instance, an English speaker who knows that VP-fronting can occur in English might quickly generalize V2 from Subject, then locative, then Object, to full V2 with any XP in German. If the English speaker learning German passed through the same stages, he might reject V2 with VP-fronting just as a child does.

A real possibility is that while children gradually proceed to add lefthand environments for V2, L2-speakers acquire OVS, then hear VP-fronting and essentially jump to the full XP-V-YP abstraction. This is potentially a very important L2 variation in the acquisition path. It would suggest a topdown bias that might have pedagogical implications. If true, it might correspond to L2 pitfalls as well: domains where too broad a generalization is introduced.

The claim that V2 is not full-blown instantly is evident in the fact that Topicalization and V3 are a known problem for V2 speakers.

## Topicalization, interfaces and V3

Our argument is, once again, that UG supplies Multiple Grammars and that critical rules are stated in Minimal terms which invites overgenerating abstractions.

Such a system seems extremely unconstrained. Therefore, we claim that constraints must be present, but from a much different source—not limitations on Feature-bundles in the syntax, nor conditions of lexical representations (e.g., verb particles). Instead we will seek Interface conditions —a view advanced by much modern research. We argue that these constraints may be language-specific. The ultimate question is not whether Interface conditions apply, but how we can state them with a precision that produces exact L2 representations.

Topicalization is an appropriate test case for this interface question. It has been observed that foreigners learning German have difficulty with V2 when Topicalization occurs, and in parallel German speakers have difficulty blocking V2 with Topics (that is producing V3) from a German perspective (see Meisel, 2011 for a literature overview):

(33) a. V3: John I like
b. V2: John like I

It is easy to see this issue in purely syntactic terms of whether V2 has applied or simply V-to-T.

Rankin provides several citations for the unsurprising claim that V3 is an L2 challenge:

“Meisel (2011, p. 132) points out that “[ungrammatical V3 order] represents a particularly persistent pattern in the speech of L2 learners of German.” This is supported by research on L1 English-L2 German by Beck (1998), who finds that learners have persistent problems with the V2 pattern. The influence of L1 word order is thus known to persist at higher proficiency levels.”

Here we shall argue that much more than the level of abstraction for XP in a V2 rule is involved.

### Information structure and strict interfaces

The status of Topicalization in a grammar has deep roots in issues of Contrast, Focus, and Exhaustivity, which link to current work on Information structure that remains both intricate, unresolved, and sensitive to language-particular variation. We discuss several offshoots before we turn to Topicalization.

Our first goal here is to pose questions which respect the potential role of this interface. Sorace (2011) suggests that L2 variation is vulnerable to indeterminacy at the interfaces and we consider this a valuable and plausible hypothesis. The hypothesis, nonetheless, should be constrained by developing a larger tapestry.

In Roeper (2014) an argument is advanced for Strict Interfaces which I argue here should be present as a backdrop to any claims about Interface variability. The claim is that certain fundamental interfaces must, quite obviously, be presupposed as universal: we assume that phonology links to syntax and syntax links to semantics. That is, humans are not parrots who can master only phonology. We suggest that at a more refined level, UG has the following constraint:

(34) UG obeys Minimal Modular Contact

That is, in the ideal case, two modules have one point of contact through which information flows, which vastly restricts the set of possible syntax-semantics-pragmatics mappings that a theory of interfaces can automatically imply. Consider the notion of Agency. It can be found in the projection of verbs in morphology (-er), projection of roles onto syntax (subject position), and via implicit arguments in the passive.

However, each of these dimensions is mediated by the verb:

(35) Verb maps AGENT onto: Subject position
Implicit Agent
-er

Therefore, -er does not carry Agency by itself, but only if licensed by a verb, which also projects Subject-Agents and implicit agents.

The point becomes clear when one considers child examples like:

(36) “I'll be the listener and you be the storier” (Maria Roeper)

which a child said, but no longer says. Why is such a handy and natural noun (storier) dropped? While –er could be identified with AGENT and therefore attach to nouns, UG demands that the AGENT-role must be linked to specific verbs and projected from the Verb—which has a single point of insertion (hence contact) in the sentence, from which it projects onto the morphology (-er), syntax (subject), and semantics (implicit argument structure). A child will drop storier when the verbal interface is built and the constraint obeyed^16^. This property of verbs as the Contact point between a dimension of semantics (Argument structure) and syntax is presumably universal.

### Imperatives

Likewise there is a natural interface for imperatives between syntax (delete you), semantics (imperative force), Pragmatics (visual situation) and stress intonation (emphatic verb). Very young children understand:

(37) “don't” [applies to child's action]

It seems natural to assume that the imperative interface is largely innate. And the connection between Contrast and Stress and the semantic projection of sets could be innate, although delayed until children have the world knowledge to project appropriate sets. Thus, the capacity to substitute for the stressed word producing different sets could easily be innate:

(38) a. Don't throw BIG STONES.
b. Don't THROW big stones

creates separate verb and noun sets. Neither the intonation pattern nor the appropriate sets are UG-fixed, but the interface among them could be. Therefore, the “variable” interface itself might be quite small, though significant nonetheless. We shall try to further reduce the variability by claiming that it is not random but reflects only grammatical choices.

### The discourse option

So where does Discourse reference belong in this realm? Information structure, primarily in terms of Givenness, has been prominently alluded to in Scandinavian studies Eitler and Westergaard (2014)^17^. We argue below that this approach needs to be enriched to include a full description of the Interface and factors like Exhaustivity and Contrast.

Work in L1 has suggested, from several perspectives (Rizzi, 2000; Yang, 2002; Hyams, 2011; and others) that if children's grammar begins in a way dominated by context and discourse, then they should allow Topic deletion (which we discuss in obviously rather simplified terms (see Sigurdsson, 2011 for some discussion). One should see if this extends to L2 speakers as well. In fact, most English speakers are not uncomfortable with discourses where subjects are deleted because they are identical to Topics (Perez et al., 2007):

(39) a. X: have you seen John anywhere?
b. Y: __went outside a few minutes ago.
while closely related forms would seem faulty:
c. Y: ^*^ __is outside.

Why? Perhaps because “outside” by itself is available. “Outside” answers a hidden question-under-discussion “is he inside “anywhere?” Maybe, re-projection of a new Question-under-discussion is preferable to an empty subject. Thus, the application of this Topic-drop principle, not a core part of English, shows subtle variation. Knowledge of such variation we might not expect of an L2 speaker. Would a German L1-English L2 speaker judge (38 b.c) the same way? Or would “__ist draussen” (is outside) be just as good for her? For me, an English L1-German L2 speaker, no clear judgment is available, but I would guess that it is more acceptable^18^.

Such issues interact with V2. Consider the environments where exhaustivity arises (see Schulz et al., 2015) for a refined discussion). Although it is not clearly universal (French and Mallayalam are reported to be exceptions), clefts imply exhaustivity in English (See Kiss, 1998; Heizmann, 2012) which children do not initially grasp:

(40) a. it was the dog that ate the cheese

(39a) implies that no one else ate the cheese. It has been suggested that Topicalization also carries exhausitivity, either via a real Operator or as a presupposition at another level:

(41) stones, John picked up.

means he picked up nothing else. However, the sentence:

(42) John picked up stones.

has a weak implicature that nothing else was picked up (Kratzer, 2009), but it certainly does not carry this as a part of its truth value.

Does this hold for grammars where Topic is more generally applied such as German? We may not have a definitive answer at the moment. Nevertheless, what should we expect of an L2 speaker coming from a Topic-dominant L1? Would both of these be grammatical without an exhaustivity expectation:

(43) Dogs, Jim likes
Dogs likes Jim

We might expect that the L2 speaker will in fact generate both options, but use the potential Information Structure difference as the basis for a choice. What could that difference be?

Let us make two simplified assumptions (whose simplicity might correspond to L2 assumptions), based on the discussion above, and then build an artificial interface which an L2 speaker might also build. We develop this idea for demonstration purposes only, not as a claim about these language families:

(44) Non-Topic oriented language:
Topicalization is: a) contrastive
b) exhaustive
Topic-oriented language:
No contrastivity: no exhaustivity
Syntactic V2

Suppose an English speaker acquiring German hears:

(45) Hunde mag Hanns [dogs likes John]

but makes no special assumptions. Then he wishes to express Contrast or Exhaustivity via Topicalization. He might then in German utilize an English device to indicate exhaustivity, saying incorrectly:

(46) Hanns mag Tiere nur selten, aber
Hunde, Fritz mag.
[Hanns likes animals only rarely, but dogs Fritz likes]

On other occasions, V2 could arise where this implication was immaterial as in (44). Thus, apparent variability at the interface could be resolved into distinct choices available to the L2 speaker applying MG, but not the monolingual speaker.

The reader can see how this toy scenario works. We do not have to assume that there is pure indecision leading to variability, but rather, at a subtler level, we apply MG theory, via an available UG interface option, which creates two options. An L1/L2 speaker uses both depending upon the interface circumstances, thus never using a “variable” grammar.

An interesting challenge here would be to design experimental scenarios that might elicit these distinctions.

(47) [scenario: John catches fish but also a turtle]
a) “Fish caught John”
b) “Fish John caught“

Now if only (46b) is exhaustive, then the L2 speaker might say (46b) in German in order to capture the exhaustivity. On other occasions where only emphasis is sought, we would find (46a).

In other situations, where the meaning is not grammatically captured, then it must be otherwise unreliably inferred. Kratzer (2009) suggests that there is a hidden equivalent of only at the pragmatic level. This approach to Interfaces claims that what looks like variability might be an effort to impose greater semantic exactitude through L2.

We can now enlarge our realm of possibilities to include this strong claim, which is useful in framing the acquisition problem even if it proves questionable:

(48) Languages may have Unique Interfaces

That is, the combination of syntax, semantics, and pragmatics might involve an implication in a particular language that is unavailable directly in other grammars (although surely communicable by more indirect means).

Suppose for instance there is an Honorific in a language and a Topic rule, such that we combine Exhaustivity with an implication that the Honored person must be present. Then we arrive at the meaning: only one such person is present now. This approach supports the intuition that while anything can be said in any language, some meanings might be grammatically expressible via grammar in one language that must be explicitly asserted in another. We can conclude that if there are unique Interfaces, then it is exactly L2 and Heritage language research which may be able to isolate them.

### Expletives and MG

While one might suppose that Topicalization rules out V2-like inversion altogether in English, this is not the case. Consider this contrast:

(49) there are three bananas.
a. Only two of them is it good to eat
b.^*^Only two of them, it is good to eat.

Expletives do not seem to allow Topicalization in English without inversion. What would the German L2-speaker of English think? Here we might imagine exactly that the syntactic availability of both forms could mislead the advanced speaker who restricted V2 for Topic, giving V3 in English, into saying or accepting V3 or (b) when that would be a mistake. This would be an example of an L2 speaker applying an overgeneral V3 rule that allowed expletive to follow a Topic.

## Missing subjects and objects

Our focus on MG and modular compatibility has focused on V2, but we will briefly note that there are two other domains where an analysis in another grammar does not disturb other modules: empty subjects and objects.

Perez et al. (2007) reports that children will misanalyse empty generic objects as discourse-linked, which is grammatical in Spanish and Portuguese. Consider this situation:

(50) Scenario: mother is cooking eggs.
Child: look Mom, I caught a fish.
Ask Subject: Is the Mom cooking__?

Spanish children and even English-speaking children initially say:

“no” because the Mom is not cooking fish, filling in the object of cook with a contextually salient object. Spanish speakers of L2 say that they must actually suppress this reading [Luiz Amaral (pc)] in order to favor a generic object [cook (something)], to which the answer is “yes” (since she is cooking eggs). Note that this case satisfies Compatibility because there is no misanalysis in another module.

Likewise missing subjects can be used by an L2 speaker without disturbing another module:

(51) Where's John = > “__is singing”

and therefore is predictable in this theory.

### Modular incompatibility

What happens where there is incompability with another module. We can generate a prediction^19^. Schouwenaars et al. (2014) report that Dutch 5 year olds will overapply SVO analysis to object-fronted sentences even when the subject-verb agreement should force an OVS reading:

(52) who wash-plural the dancers
= > who are the dancers washing t

This result might be found among L2-speakers or via eye-tracking which would indicate that an SVO analysis operates at a superficial level and then undergoes revision as new modules are added. This interaction among modules might well be most visible via research on L2. It could lead to quite subtle degrees of acceptability.

### Minimalism and abstraction

A general consequence of Minimalism is that rules are stateable at a very abstract level. One can, for instance, build structures with decisions about Labels left partly open. This creates extra L2 room for uncertainty^20^.

Where else can we find evidence of the abstract level of a rule? Here is a case one might subject to greater scrutiny. In English we find many speakers (including me) who say things like (from a Google for “could have I/you”):

(53) “How could have I passed the exam”
“How could have you done this to me?”
“how could have you used your powers for evil?”

instead of:

(54) How could I have passed the exam

Will an L2 speaker allow both in comprehension or production? The answer most probably lies in whether the grammar represents inversion with an Aux-Head or an unspecified AuxP:

(55) NP AUXP/AUX V

This is an empirical question, but if the approach advocated here is correct, then speakers should aim for more abstract representations rather than less abstract ones. Therefore, the AUXP inversion will probably not be rejected so easily by L2 speakers, even if not used.

In a sense we can characterize the L2 acquisition path as topdown rather than bottom up. If the child builds up a very narrow range of possible environments initially and finally generalizes to a full range of invertable Auxiliaries, the L2 speaker might seek to build the most abstract form as quickly as possible as an instance of representational economy.

### Dialects and compatibility

Is it impossible to write features of one grammar into another? Green and Roeper (2007) argued that one way to define a dialect is in terms of Tree-compatibility. Green has argued that there is an Aspectual node in African-American English. It can be added between IP and VP in Mainstream American English without disturbing the tree, but with discernible consequences:

(56) a. He be playing baseball, don't he.
b. ^*^He be playing baseball, ben't he/isn't he.

Here we find the tag-question indicates that the habitual be belongs to an extension of VP, not IP, therefore requiring do-insertion, just like a Main Verb. Therefore, it must belong to the Verb-projection, not IP:

(57) He plays baseball, doesn't he.

Non-AAE speakers understand and sometimes use Habitual BE, but fail to form do-tags, suggesting that they assimilate it to IP and not VP. In any case, the dialect speaker who also controls the Mainstream form will need to have diacritics to indicate social factors that dictate whether the extra node should be allowed.

### Variational learning, feature-reassembly, full T/full A

In a sense, the MG approach is a methodological proposal orthogonal to, not in opposition to, current theories. The essential proposal is simply to formulate the grammars of L2 with sufficient technical precision that they predict what is ungrammatical in the manner of L1 research. To capture the formal “variability” one should state as rules or grammars the options selected. Of course, whenever formal variability arises, it invites myriad social and pragmatic factors to participate, producing the surface variability of sociolinguistic “optionality,” which—if we understood them fully—may or may not be represented as Features in the formal rules.

#### Variational learning (VL)

The VL approach has MG as a prerequisite^21^. Yang (2002) argued that each side of a parameter—both of which must be present—is linked to a probability which is increased or decreased by further evidence. It could not exist if one attempted to represent the facts within one grammar with complex exceptions. The unchosen—or non-productive side remains in an available grammar.

Yang argues that the weight on one side of a parameter over another is increased or decreased in terms of input experience. An interesting question to ask here in this light is whether one is responding primarily to types or tokens.

Consider the pro-drop parameter which is arguably triggered by sufficiently frequent exposure to one type, there-insertion. When a child hears enough examples of it^22^, then English is represented as -Pro-drop. However, many, many examples, like:

(58) a. seems nice
b. looks good.

exist so that the +pro-drop parameter seems to survive linked to specific verbs, no matter how frequent they are.

In the case of V2, as we have discussed, it is the types of constructions which can occupy the lefthand XP position which seem to be critical to the eventual productivity of the expression.

Nevertheless, the English speaker also operates with verb classes so that the fairly large class of speaking verbs uniformly permits it:

(59) “nothing” roared/muttered/sighed/moaned Bill

And the verb be is extremely productive and compatible with V2. We say:

(60) a. How is it
and not:
b. ^*^How do it be.

A brief search in CHILDES revealed 6 children who appear to generalize this to the category of equatives and say:

c. “what means that”

or a period before it is eliminated by hearing “what does that mean.” The large number of be sentence tokens, however, does not trigger generalized V2 as in German. Therefore, the type/token difference is important. We do not yet know how to conceive of the balance between them in order to determine productivity. Is it the many types or many tokens which etch a rule into a grammar?

#### Feature-reassembly

Another approach is to reduce all variation to features which can then be variously valued as proposed by Feature-Reassembly (Lardiere, 2009) who provides insightful efforts to apply modern linguistic distinctions to L2. Often it is not exactly clear where the weight should fall: feature choice, uninterpretability, feature-assembly, morphology, or meaning variation. Lardiere (2009) shows quite well how the theories of parameters and micro-parameters overgenerate, providing an insurmountable range of options, and do not make precise predictions. We agree with her apt summary:

“Parameter-setting, however, has never coped very well with the issue of variability, which is often a persistent hallmark of second language development. (By “variability,” I mean here the variable omission, underspecification, overreliance on default forms, and/or apparent optionality vs. obligatoriness of the morphophonological expression of grammatical properties.) As van Kemenade and Nigel (1997) point out, since parameter settings are typically all-or-nothing phenomena, the resetting of a parameter should represent an “abrupt change” in a speaker's language (p. 4). The persistence of observed variability in the acquisition data is thus not predicted, insofar as the presence or absence of some grammatical property should be tied to the learner's having set the plus or minus value of a particular parameter.”

This critique leads us precisely to think that we need a conception of L2 and language variation that is pitched at principles expressed at the macro-level: Head direction, wh-movement, LF variation. Once these choices are formulated as independent grammars, which are all present in everyone's UG, variability follows naturally (even if conditioning social, phonological, and pragmatic factors are difficult to state).

What is being proposed here is not in opposition to these potentially useful notions from Feature-reassembly which appropriately argues that feature addition and subtraction are insufficient. Rather, once again, MG offers a different methodological approach. It suggests that L2 research, whatever mechanism and formalism is involved, should proceed from exact formulations that arrive at predictions of acceptable or unacceptable grammaticality for an L2 speaker. This is how generative grammar began: very simple, now almost quaint formalisms in early work by Chomsky, but a steady refinement of them with predicted and rejected instances of acceptability/grammaticality. Without a sharp edge of this kind, I believe it will be difficult to build the kind of theory of L2 acquisition that most researchers would like to see.

Thus, to capture variation at that level, particularly that which reflects both L1 and L2, we need to write out two independent grammars and claim that they are both active. In order to do that, one needs particularly abstract representations—exactly of the sort we have been discussing. Access to the abstraction as a starting point is critical.

If one can write the grammar with abstract notions like Maximal Projection (XP), then one can begin to state the variations as we have done above:

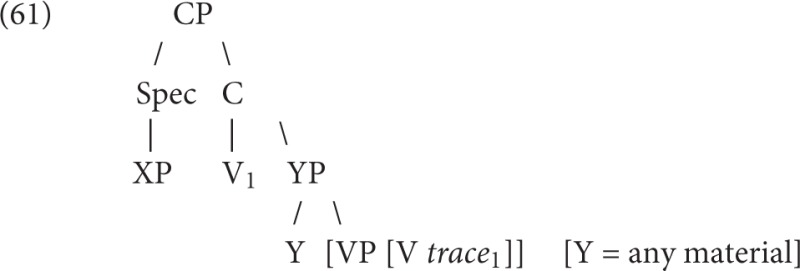

where VP can allow variation under a modified Head Constraint to include V-particle, or Aux-Head and AUX-complex.

We need a perspective more abstract than Feature-reassembly to capture this. Consider the prediction Lardiere (2009) makes that a Chinese speaker confronted with “I bought fruit” will give a Real question answer rather than an Echo-question:

(62) a. A. “I bought fruit”
B. “you bought what” = echo-question = >
A.“I bought fruit”
b. A. “I bought fruit”
B. “you bought what”
= Real question, seeking specification
[ = “what did you buy”] ⇒
A. “I bought bananas.”

(b) asks for greater subdivision of given knowledge, a subset answer. Why this prediction? It is clear from her results that both options are available, so we need to state them both as alternatives.

#### Full access/full transfer

The original proposal of FA/FT by Schwartz and Sprouse (1996) launched a tremendous amount of detailed work seeking every hint of cross-linguistic effects in the L2 process. This large net is a natural first step and very valuable. However, the concept seems to presuppose that one inserts part of a grammar into another with a great deal of minute adjustments then following and perhaps a great deal of L1 baggage that does not fit. FA/FT does not have a natural way to capture the dual analysis of particular simple sentences like those cited from Rankin: who woke John up. And it does not have a metric to describe the diverse impact of different levels of grammar. Perhaps one should see MG as beginning to carve out a space for such metrics which could reflect Interface boundaries (as Sorace suggests), although we regard much of the interface domain to be universally determined and precisely where little variation occurs.

To appreciate one case where Transfer is examined, consider Özçelik (2009) who looks at Inverse Scope in Turkish:

(63) a. Donald didn't find two guys = inverse two guys>not
[ = there are two guys Donald did not find]
surface: not> two guys
[Donald did not find (any) two guys]

The author comments:

“intermediate English L2ers should behave noticeably worse than advanced English L2ers due to the initial transfer of the Turkish setting, as well as the ongoing acquisition of the L2 setting. However, our intermediate English L2ers did not particularly do bad enough to be qualified as “transferring from the L1.”

Inverse scope is a major option in the organization of LF and therefore we would expect it to be among the abstract rules that is available as a separate entity from UG with minimal triggering required. If we can assume, therefore that if they have any evidence that invites Inverse Scope, then that grammar will make Inverse Scope available and it can apply. It is not a question of whether it came from Turkish or whether it is transferred to English, but simply whether evidence has arisen to instantiate that important UG option. Once present we would expect it to remain as a comprehension option even if speakers were able to avoid it in production. The Comprehension/Production distinction is particularly important for L2 (see Amaral and Roeper, 2014).

#### Typological primacy model and bottleneck theory

Rothman's (2013) presentation of the Typological Primacy Model shares much with our approach, in particular the desire to make strong predictions and to argue that what is transferrable depends upon the grammatical status of constructions. Generating predictions strong enough to be proven “wrong” is the traditional path to refinement in the history of generative grammar.

While the TPM puts an emphasis on the intertwined nature of syntax, morphology, and phonology, we argue that it is exactly the extent to which properties of a given module can be cast in an abstract independent form—be un-entwined—that will dictate their transferability. In that light, as Rothman points out, LF transfer works cross-linguistically. He also cites Özçelik (2009): “[who] argues explicitly for and shows convincing evidence of overall typological and not property level structural transfer in line with the TPM, showing that Uzbek–Russian bilinguals of L3 Turkish transfer scopal properties of Uzbek, a Turkic language like Turkish, despite the fact that Uzbek works differently and Russian and Turkish are identical in this regard.”

Likewise his notion of “degree of similarity” refers ultimately to the degree to which different modules are intertwined, so that the less other modules are involved, the more likely a simple, transferrable rule is possible. The TPM refers to this notion as “non-facilitative” which is the same prediction our account makes. The challenge, as always, is to define sharp representational options for what is claimed.

We can extend the LF example further in terms of interaction with case-marking. If case-marking is universal, but can be abstract and show no morphological effect, as is generally the case in English, then it will not interfere with LF formation, therefore Transfer should occur.

(64) someone loves everyone = > LF [everyone > someone]

But note that the theory could be shown to be wrong if one language shows no case-Marking while another language marks quantifiers with case, like German:

(65) Jemand liebt jeden [object-case marking] (someone loves everyone)

If then transfer to or from German with case-marking of Scope inversion is more difficult than transfer to Chinese without case-marking, then it would show that LF does not have modular independence. And if a language marks both nominative and accusative on quantifiers, then we might predict even less LF transfer. If so, then we would have evidence of interference, presumably blocking use of LF scope inversion. But again, if LF has a case-independent representation, then it should transfer in all languages equally. These are, clearly, easily approachable empirical questions.

Moreover, this example may be a domain where we can fulfill the promise that cross-linguistic comparative work can further articulate UG. It is safe to say that there is a common intuition that LF movement has nothing to do with case-marking: we do not have to move invisible case-marking when we covertly move a quantifier. If there is no contrast between LF in case-marked and non-case-marked languages for transfer, then it is direct evidence for this intuition, which should ultimately be stated in a fully-articulated representation of UG.

Consider now Bottleneck theory. Slabokova (2014) argues that the involvement of Functional Categories (FC) proves difficult to transfer across languages. Again the generalization implicitly refers to the fact that FCs (e.g., CP) can engage other modules, like wh-movement. If we have a sentence like:

(66) Whom did you talk to__

We have not only the projection of CP, but a Question-Probe feature which causes wh-movement to occur, but only after case-marking has applied. The fact that several modules are involved is doubtless related to the fact that case-marking is weakening in this construction and allowing who did you talk to_ for many speakers. On the other hand, direct lexical expressions of FC's (like complementizers that or to) may show minimal transfer inhibition or delay in acquisition. Once again we argue that it is the interaction of several modules that may block easy application of one grammar inside another as it is formulated in MG. Such interactions may be very common in FC's, but it may not be the concept of FC itself which is the source of difficulty.

## Conclusion

Let us summarize our approach. The MG theory is, in a basic sense, an inevitable consequence of the abstract nature of modern minimalism. It means that via abstraction one can state common rules across many grammars. This is a more powerful UG claim than the traditional view that the building blocks of all grammars are identical.

Another emphasis in this essay is that many of the MG options remain at an abstract level and are constrained by unique interface restrictions rather than restrictions stated on the rule itself. Our goal has been to propose that if we articulate full MG options that include fixed Interface representations, avoidance of other modules that complicate the application of rules, we will have a method to generate more precise acceptability/grammaticality judgments from L2 speakers. In this approach, the notion of Transfer is supplanted by explicit presence of two analyses whose status can be experimentally explored.

It follows naturally that if we allow ourselves more abstract representations, then those representations lend themselves to the idea that a rule can apply across grammars, or that alternative rules (V=>Tense, V=> Comp) are jointly available for both monolingual and bilingual speakers. These questions can be approached applying detailed experimental apparatus, which we have presented here in a speculative manner. Altogether, it should be clear that a whole phalanx of predictions arise from the MG account.

This leads to what might seem like a paradoxical result. Although one might say that the presence of two grammars should make analysis more obscure and ambiguous, the argument here is that it is precisely this assumption, used in L2 research, which can isolate fundamental properties of grammar where monolingual analysis permits too many alternatives to make a decisive choice. If successful, then research on multi-lingualism holds the promise of theoretical insights unobtainable anywhere else.

### Conflict of interest statement

The author declares that the research was conducted in the absence of any commercial or financial relationships that could be construed as a potential conflict of interest.

## Footnotes

^1^Thanks to Luiz Amaral, Leah Bauke, David Erschler, Stefan Keine, Terje Lohndal, Tom Rankin, Marit Westergaard, Rebecca Woods, the class on Variation with Lisa Green, and Frontiers reviewers for helpful discussions.

^2^This essay is written by someone who works primarily in L1 acquisition. It is important that discussions which cross boundaries between close disciplines are promoted. One consequence is that it is virtually impossible to be familiar with the whole voluminous L2 literature and therefore relevant arguments and evidence may exist of which I am unaware or unable to explore carefully. I would be happy to receive any information relevant to both the theoretical and empirical claims advanced here by those with more detailed knowledge. The broad focus on FA/FT, Feature-Reassembly, and Variational Learning I take to be a fair sample of currently explored approaches, but I welcome further detailed information pertinent to the claims in this paper, which may have easily escaped my attention.

^4^FT/FA hypothesizes that the initial state of L2 acquisition is the final state of L1 acquisition (Full Transfer) and that failure to assign a representation to input data will force subsequent restructurings, drawing from options of UG (Full Access). By contrast, our approach suggests that simple rules allow the grammars to remain present as distinct entities, producing unexpected ambiguities. The idea of Minimal trees suggested by Vainikka and Young-Scholten (1996) is very much in the spirit of seeking the minimalist substructure that can transfer, but we do not argue that more refined features cannot have an impact.

^5^In Roeper (1999) and Amaral and Roeper (2014) for MG and L2.

^6^We take the term module to include the traditional ones: Case, Agreement, Move-alpha, LF-formation, although some recent theories suggest that modules can be replaced by interface requirements, this seems premature.

^7^See Lohndal (2013) for background discussion.

^8^Leah Bauke (pc) points out that inherent reflexives block Topicalization as well:

i. ^*^ Sich hat Hanns mit Maria gestritten.

This falls into line as well as an example which fails to be a Maximal Projection or have an acceptable Label.

^9^Müller (2003) is an edited volume where a common theme is that CP is a “vulnerable domain” in L2 and in disorders. This also seems to suggest that that it is linked to an Interface and therefore, perhaps, more easily subject to alteration or error. In the case at hand, it would have to explain the opposite—a tighter requirement on verb-raising which involve necessarily moving the particle as well.

^10^It is not linked to length. A monosyllable is also excluded: ^*^ I hollered “no” out.

^11^All the corners should be explored. Consider incorporation which also blocks incorporation from a stranded particle (ii) stranding:

i. ??paper-up-picking
ii. ^**^ up-paper-picking

See Bauke and Roeper (2011) for a morphological analysis in terms of Labeling theory. One might predict that a German L1 speaker would find (i) more acceptable because particle verbs in German allow incorporation.

^12^As I wrote this article I realized that I was carrying out an implicit experiment on myself. My L2 knowledge of German is regarded as very good and I trust myself to create examples. However, when seeking to establish that this constraint did not apply to German, I started to feel uncertain about (ii), although I suspect it would earn a strong ^**^ from a native speaker and not a “?,” but I cannot tell:

i. Er liest mir das Buch aus
ii. ?Er liest mir aus das Buch

One might presume that it is contemplation as a linguist which is the source of my uncertainty, but I suspect that it would be widespread and would deserve a careful study together with all the facts mentioned here (see Rankin, 2013 and references therein).

^13^Thanks to Leah Bauke for discussion.

^14^Bruening (2013) suggests that phonological parallelism constraint is present to capture facts about quotation, but it does not deal with facts like these which clearly belong to the puzzle.

^15^In fact, it seems very likely to me that OVS is acquired before 4 years, but the argument remains the same: the rarer elements specifying the left hand variable will not be added all at once, as the Topic example below illustrates.

^16^Note that nouns can take –er: New Yorker, Detroiter while other nouns take –or (donor), and many idioms arise sinker (see Roeper (2014) for discussion), but their readings are idiosyncratic. See Bauke and Roeper (2011) for discussion of where compositionality in the lexicon can be found systematically.

^17^Eitler and Westergaard (2014) argue that the choice of V2 vs. non-V2 in OE/ME was dependent on information structure: The word order XSV (non-V2) was preferred if the subject was informationally given (often a pronoun) and XVS (V2) if the subject was focused or new information (often a full DP).

^18^Hyams (2011, p. 42) recounts a number of L1 studies that show Discourse sensitivity: “Allen (2000) shows the argument omission vs. overt expression can be significantly predicted by the degree of “informativeness” of an argument (as measured by several variables including newness, contrast, absence, differentiation in context and person). Serratrice and Sorace (2003), using the same principles introduced by Clancy and Allen, also find significant discourse/pragmatic effects in the distribution of overt vs. null subject in six Italian-speaking children (ages of 1:8 and 3:3), reflected the distribution of the adult language. Serratrice and Sorace are explicit in assuming that the pragmatic principles operate within the boundaries imposed by the grammar, in this case a pro-drop grammar.”

^19^There may well be relevant data available on this question of which I am not aware.

^20^Here is typical anecdotal case of L2 leaving a wh-node without a feature. A foreign student once asked whether whose in English must be a person because it has who within it which by itself does have this restriction. In fact, of course, it is not required, but the evidence for this may not arise everyday and therefore one would seem entitled to continue the assumption that the morpheme who had a [+person] restriction.

^21^See Kroch and Taylor (1996) for connections to historical grammar and the earliest Multiple Grammar proposals for generative grammar. As one reviewer suggests, the approach should naturally apply to intermediate stages in the history of grammar. See Yang and Roeper (2011) for a broad background.

^22^See Hyams (2011) for a current overview which also articulates other dimensions besides a single trigger. While other factors may support or be a pre-requisite, it is not clear that a few central triggers are not the basic pivot around which a parameter is set. See Holmberg (2010) for a sophisticated presentation along these lines.
